# Adipose tissue pathways involved in weight loss of cancer cachexia

**DOI:** 10.1038/sj.bjc.6605665

**Published:** 2010-04-20

**Authors:** I Dahlman, N Mejhert, K Linder, T Agustsson, D M Mutch, A Kulyte, B Isaksson, J Permert, N Petrovic, J Nedergaard, E Sjölin, D Brodin, K Clement, K Dahlman-Wright, M Rydén, P Arner

**Affiliations:** 1 Department of Medicine, Karolinska Institutet at Karolinska University Hospital, Stockholm SE-141 86, Sweden; 2 Department of Surgery, Karolinska Institutet at Karolinska University Hospital, Stockholm SE-141 86, Sweden; 3 Department of Human Health and Nutritional Sciences, University of Guelph, Guelph, ON, Canada N1G 2W1; 4 The Wenner-Gren Institute, The Arrhenius Laboratories F3, Stockholm University, Stockholm SE-106 91, Sweden; 5 Department of Biosciences and Nutrition, Karolinska Institutet, Stockholm, Sweden; 6 INSERM, U-872, Nutriomique (team 7), Paris 75006, France; 7 University Pierre and Marie Curie-Paris 6, Cordeliers Research Center, Paris 75006, France; 8 AP-HP, Pitié-Salpétrière Hospital, Paris 75013, France

**Keywords:** adipose tissue, wasting, microarra*y*, cellular adhesion, extracellular matrix

## Abstract

**Background:**

The regulatory gene pathways that accompany loss of adipose tissue in cancer cachexia are unknown and were explored using pangenomic transcriptome profiling.

**Methods:**

Global gene expression profiles of abdominal subcutaneous adipose tissue were studied in gastrointestinal cancer patients with (*n*=13) or without (*n*=14) cachexia.

**Results:**

Cachexia was accompanied by preferential loss of adipose tissue and decreased fat cell volume, but not number. Adipose tissue pathways regulating energy turnover were upregulated, whereas genes in pathways related to cell and tissue structure (cellular adhesion, extracellular matrix and actin cytoskeleton) were downregulated in cachectic patients. Transcriptional response elements for hepatic nuclear factor-4 (HNF4) were overrepresented in the promoters of extracellular matrix and adhesion molecule genes, and adipose HNF4 mRNA was downregulated in cachexia.

**Conclusions:**

Cancer cachexia is characterised by preferential loss of adipose tissue; muscle mass is less affected. Loss of adipose tissue is secondary to a decrease in adipocyte lipid content and associates with changes in the expression of genes that regulate energy turnover, cytoskeleton and extracellular matrix, which suggest high tissue remodelling. Changes in gene expression in cachexia are reciprocal to those observed in obesity, suggesting that regulation of fat mass at least partly corresponds to two sides of the same coin.

Cancer cachexia is a life-threatening condition that is typically seen in half of untreated cancer patients (Fearon and Moses, 2002; Deans and Wigmore, 2005). It is associated with a specific loss of adipose tissue and skeletal muscle mass, decreased survival and poor response to chemotherapy (Esper and Harb, 2005). Although hyper-metabolism without a compensatory increase in food intake is common in these patients, these factors can only partly explain the loss of weight (Bosaeus *et al*, 2001).

The mechanisms behind tissue loss in cancer cachexia are unknown, but alterations in gene expression could be involved. Increased lipolysis and fatty acid (FA) oxidation and changes in expression of corresponding regulatory genes have been shown in adipose tissue of patients with cancer cachexia (Agustsson *et al*, 2007; Laurencikiene *et al*, 2008). Systemic inflammation is found in human cachexia; however, adipose tissue levels of cytokines, and leukocyte and macrophage markers are not altered (Ryden *et al*, 2008). In muscle of cancer cachexia patients increased expression of genes regulating the ubiquitin–proteasome pathway has been observed (Williams *et al*, 1999; Bossola *et al*, 2001).

Gene expression profiling is a useful tool to identify pivotal regulatory pathways for altered function of adipose tissue (Clement *et al*, 2004; Dahlman *et al*, 2006). In this study we used global gene expression profiling to identify molecular pathways associated with weight loss in cancer cachexia. We investigated patients with gastrointestinal cancer who had no evidence of gastrointestinal obstruction, and were either weight stable or with evident cachexia. We focused on adipose tissue as preferential wasting of this tissue is involved in the early stages of disease (Fouladiun *et al*, 2005). Our primary hypothesis was that changes in pathways involved in the regulation of energy metabolism would accompany loss of adipose tissue in cachexia. The change in fat mass observed with obesity is opposite to that seen with cachexia. Our secondary hypothesis was that this reciprocal change in fat mass is mirrored at the level of gene expression. The latter question was investigated by comparing global gene expression changes in the adipose tissue of cachexia patients with our previously generated findings examining obese and lean subjects (Dahlman *et al*, 2005; Mutch *et al*, 2009).

## Materials and Methods

### Patients

Patients scheduled for gastrointestinal cancer operation between March 2004 and March 2008 were evaluated for the study and patients who (a) were able to come to our clinical research laboratory for an adipose tissue needle biopsy and relevant clinical examinations in spite of their cancer, (b) had not received prior anticancer treatment, (c) did not have clinical evidence of gastrointestinal obstruction, (d) fit into the logistical scheme for scientific studies before operation or preoperative treatment and (e) were willing participants were included (*n*=53). None of the selected patients had jaundice. The study was approved by the Regional Ethics committee. The investigation was explained in detail to each patient and written informed consent was obtained. The patients were divided into two groups on the basis of diagnosis after surgery. The first group of patients had cancer cachexia (*n*=13), which was defined as gastrointestinal cancer with self-reported unintentional weight loss of >5% of the habitual weight during the previous 3 months or >10% unintentional weight loss during the previous 6 months (Agustsson *et al*, 2007). The primary location of cancer was pancreas (*n*=11), stomach (*n*=1) and colon (*n*=1). The second group (*n*=14) consisted of patients with gastrointestinal cancer with no significant self-reported weight change during the last year. The localisation of malignancy was pancreas (*n*=6), oesophagus (*n*=1), stomach (*n*=1), colon (*n*=3), gall bladder (*n*=1) and liver (*n*=2). The remaining 26 patients were excluded because (a) although pre-diagnosed with gastrointestinal cancer they did not have a malignancy according to final histological evaluations (*n*=8) or (b) we did not obtain adequate amounts of adipose tissue, at least 0.9 g, for a complete investigation.

We used the method by Liu to determine the appropriate sample size for microarray experiments (Liu and Hwang, 2007) (see Supplementary Information for details).

We compared the changes in adipose tissue gene expression associated with cachexia to changes in gene expression associated with obesity previously reported elsewhere (Dahlman *et al*, 2005; Mutch *et al*, 2009). One study included Affymetrix HGU95Av2 expression profiles on 17 non-obese and 20 obese healthy Swedish women (Dahlman *et al*, 2005). The other study described Agilent 4 × 44 K gene expression profiles in needle-aspirated abdominal subcutaneous adipose tissue biopsies from nine obese and nine lean age-matched subjects (Mutch *et al*, 2009). The 4 × 44 K array platform comprises 41 000 unique 60-mer oligonucleotide human sequences and transcripts. mRNA levels in relation to obesity were confirmed in subcutaneous abdominal adipose tissue biopsies from non-obese (*n*=13, age 40±13 years, BMI 24±2 kg m^−2^) and obese (*n*=19, age 41±8 years, BMI 44±4 kg m^−2^) women.

To determine whether differences in gene expression detected in the groups described in the previous paragraphs was likely to have occurred in stroma vascular cells or in adipocytes, differences in gene expression between these cellular fractions were examined in subcutaneous adipose tissue in another group of overweight or obese women (*n*=9), as previously described (Clement *et al*, 2004). The cDNA microarray platform used to generate this data set was produced at Stanford University and consisted of PCR-amplified cDNAs printed on glass slides, with 42 786 spots representing 29 308 UniGene clusters. These previously published microarray studies were approved by the local Ethics Committee of the Karolinska Institutet and in France by the CPP, Hôtel-Dieu hospital (Paris). Signed informed consents were obtained from all subjects.

### Clinical examination

Patients came to the laboratory for one clinical examination after an overnight fast. Height, weight, body composition by bioimpedance using Quad Scan 4000 (Bodystat LTD, Isle of Man, British Isles) and indirect calorimetry using Deltatrac (Datex-Engstroms, Helsinki, Finland) were determined. A venous blood sample was obtained for measuring lipids, glycerol, FAs, albumin, sensitive CRP and IL-6, as previously described (Agustsson *et al*, 2007; Ryden *et al*, 2008). Nutritional status was assessed by using a standardised questionnaire for oncology termed Subjective Global Assessment (SGA) (Ottery, 1996). Tumour stage was classified post-operationally, as described previously (Agustsson *et al*, 2007). To indirectly assess lipolytic activity, *in vivo* glycerol and FA concentrations were divided by body fat weight (Agustsson *et al*, 2007).

### Fat biopsies

After clinical examination an abdominal subcutaneous fat sample was obtained by needle biopsy, as previously described (Kolaczynski *et al*, 1994). Tissue pieces were rapidly rinsed in saline. A small portion was immediately used for determination of mean fat cell volume and total number of fat cells in the body, as previously described (Agustsson *et al*, 2007). Three 300-mg tissue pieces were frozen in liquid nitrogen and kept at −70°C for global gene expression profiling, real-time quantitative PCR (RT-qPCR) and western blotting. Tissue pieces removed and frozen in this manner were free of damaged cells and blood (Ryden *et al*, 2001).

### RNA preparation

WAT (300 mg) was disrupted mechanically and RNA was extracted using the Nucleospin RNA11 kit (Macherey-Nagel, Duren, Germany). RNA samples were treated with RNase-free DNase (Macherey-Nagel). RNA concentration and purity (in relationship to protein) were measured spectrophotometrically using a Nanodrop ND-1000 Spectrophotometer (Thermo Fisher Scientific Sweden, Gothenburg, Sweden). Purity was measured by dividing absorbance at 260 nm (RNA) by absorbance at 280 nm (protein). All ratios were above 2.0. High-quality RNA was confirmed using an Agilent 2100 Bioanalyzer (Agilent Technologies, Santa Clara, CA, USA).

### Affymetrix analysis

From non-degraded high-quality total RNA we prepared and hybridised biotinylated complementary RNA to Gene 1.0 ST Arrays, and then washed, stained and scanned the slides using standardised protocols (Affymetrix Inc., Santa Clara, CA, USA). The Gene 1.0 ST Arrays measure the expression of 28 869 transcripts. Subsequent data analyses were performed using the Affymetrix GeneChip Operating Software (GCOS) version 1.4. To allow comparisons of transcript levels between samples, all samples were subjected to an all-probeset scaling-to-target signal of 100. Our microarray data have been submitted to GEO in a MIAME-compliant format (GSE20571).

### Quantitative real-time PCR

One microgram of RNA was reverse transcribed using the Omniscript RT kit (Qiagen GmbH, Hilden, Germany) and random hexamer primers. RT-qPCR was performed using the SYBR Green-based technology. A direct comparative method was used for data analysis (Applied Biosystems, Foster City, CA, USA) using 18S as internal control.

### Quantification of mtDNA copy number

The ratio of mitochondrial DNA (mtDNA) to nuclear DNA reflects the tissue concentration of mtDNA per cell and was determined by quantitative RT-qPCR as described (Bogacka *et al*, 2005) (see Supplementary Information for details).

### Protein expression

The protein levels of the adenine nucleotide translocator (ANT) and cytochrome-*c* oxidase subunit-4 (COX-4) in adipose tissue were measured by western blotting using antibodies from Santa Cruz Biotechnology (Santa Cruz, CA, USA; see Supplementary Information for details).

### Statistical analysis

Differences in adipose tissue expression of individual genes between cachectic and weight-stable cancer patients were analysed using significance analysis of microarrays (SAM) (Tusher *et al*, 2001). Quantitative analysis of weight loss in SAM produced similar results, but as the study was designed as a case–control study we focused on the latter analysis. To evaluate the potential functional importance of cachexia-regulated genes, we analysed the enrichment of gene expression within predefined biological pathways and gene ontologies (GO) in either cachectic or weight-stable patients using the FunNet, Gene Ontology Tree Machine (GOTM), and Gene Set Enrichment Analysis (GSEA) methods (Ashburner *et al*, 2000; Zhang *et al*, 2004; Subramanian *et al*, 2005; Prifti *et al*, 2008). Up- and downregulated genes were analysed separately. The FunNet tool has been extensively described elsewhere (Prifti *et al*, 2008). A false discovery rate (FDR) of 5%, which takes into account tests for multiplicity, was used to identify the Kyoto Encyclopedia of Genes and Genomes (KEGG) biological pathways overrepresented in gene expression data. GOTM compared the lists of up- and downregulated genes in cachexia with an FDR of 5%, with a reference gene list comprising all genes on the Gene 1.0 ST Arrays, and reported those GO that were statistically enriched in either cachectic or weight-stable control subjects (Zhang *et al*, 2004). GSEA orders measured mRNAs according to their differential expression between two classes, for example, cachectic *vs* weight-stable control patients, into a gene list (*L*) (Subramanian *et al*, 2005). Subsequently, GSEA evaluates whether an *a priori* defined set of genes shows statistically significant, concordant differences between two biological states. We analysed the curated functional gene sets, C2, and GOs available in the GSEA database. We used PAINT to evaluate if specific transcriptional regulatory elements (TREs) were overrepresented in the promoter regions of cachexia-regulated genes using all genes on the microarray as the reference and an FDR of 20% (Vadigepalli *et al*, 2003). We analysed 2000 base pairs upstream from the transcriptional start site according to Ensembl for TREs applying a core similarity of 1.0. An unpaired Student's *t*-test was used to analyse clinical phenotypes and the expression of individual genes. When necessary, the analysed phenotype was log-transformed to achieve a normal distribution. SGA and tumour stage were analysed with a Mann–Whitney test. Data are presented as means±s.d. or means±range.

## Results

### Clinical findings

The clinical results are shown in Table 1. BMI and body fat mass were markedly decreased in subjects with cancer cachexia. Age, gender distribution and lean body mass did not differ between the two groups. The cancer cachexia group had a tumour stage of 3 (1–4) *vs* 4 (0–4) in the weight-stable group (*P*=0.04). The cachectic patients self-reported a marked (mean value about 10%) decrease in their habitual weight. Cancer cachexia was accompanied by signs of systemic inflammation, that is, elevated CRP and IL-6. Lipid mobilisation and oxidation were increased in cachexia as evidenced by changes in the plasma concentrations of glycerol and FA, and in the respiratory quotient; however, total resting energy expenditure was similar in both the groups. Subcutaneous fat cell volume was markedly decreased in cachexia, but there was no change in total body fat cell number between the groups.

### Expression of matrix, cytoskeleton and metabolic gene sets are regulated by cachexia

A total of 364 genes were downregulated and 61 genes upregulated in the abdominal subcutaneous white adipose tissue (WAT) of cachectic *vs* weight-stable patients when using an FDR of 5% (Supplementary Table 1). Seventy-one genes were downregulated and five genes were upregulated in the cachectic patients with an FDR of 1% (Table 2). Table 2 also shows that cachexia-regulated genes were fairly evenly distributed between the adipocytes (*n*=28) and the stroma vascular fraction of adipose tissue (*n*=22). We confirmed up- and downregulated expression of genes in cachectic *vs* controls patients by RT-qPCR (Table 3).

**Figure 1:**
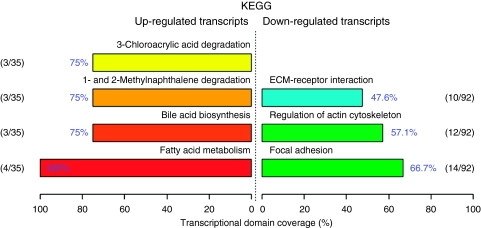
Functional profiling depicts significantly overrepresented KEGG pathways on the basis of differentially expressed genes in cachexia *vs* weight-stable patients. Using the FunNet tool, functional profiling was performed using an FDR of 5%. Only genes that have at least one annotation in KEGG were analysed. Of the 61 upregulated genes, 35 were annotated. Of the 364 downregulated genes, 92 were annotated. The number of genes associated with each significant pathway is indicated in relation to the total number of annotated genes in our lists of differentially expressed genes. Transcriptional domain coverage indicates the most significant biological functions that are represented among annotated genes, ranked by percentage.

We next investigated if cachexia-regulated genes belonged to specific pathways or GOs. As minor changes in the expression of many related genes can have a significant effect on the function of a biological pathway, we used the differentially expressed gene list obtained using an FDR of 5% (i.e., the 425 genes regulated by cachexia) (Mootha *et al*, 2003). The FunNet analysis revealed that genes downregulated in cachexia were overrepresented in pathways related to the extracellular matrix, actin cytoskeleton and focal adhesion (Figure 1), while the pathway for FA metabolism was overrepresented among the genes upregulated in cachexia. In the four upregulated pathways a core group of genes appears in all the pathways: ADH1A, ADH1B and ADH1C. The ‘fatty acid metabolism’ pathway includes an additional gene, GCDH. According to GSEA, pathways related to metabolism were upregulated among cachectic patients, for example, electron transport, FA degradation, oxidative phosphorylation and Krebs TCA cycle (Table 4). The pathways downregulated in cachectic patients are more difficult to group on the basis of name; however, on closer inspection these pathways contain many genes related to cellular adhesion and extracellular matrix. Similar results were obtained with GOTM (Supplementary Table 2). Those genes regulated by cachexia that correspond to the following canonical pathways are shown in Supplementary Table 3: cell adhesion, extracellular matrix, actin cytoskeleton, mitochondrion and electron transport. We explored potential transcriptional regulators of these pathways. In the promoter regions of the extracellular matrix and cell adhesion genes there was significant overrepresentation of the TREs for Hepatic nuclear factor-4 (HNF4) (adjusted *P*<0.0001, FDR 20%) (Supplementary Figure 1). The TREs for HNF4 were present in 20 of 51 genes. On the microarrays HNF4 mRNA levels were downregulated in the adipose tissue of cachectic *vs* weight-stable patients (146 *vs* 166 AU, *P*=0.01). No other TREs were overrepresented when accounting for multiple testing. For actin cytoskeleton, mitochondrion and electron transport no common TREs were overrepresented in the promoter regions (results not shown).

Another level of control on gene expression is mediated by co-regulators. Peroxisome proliferator-activated receptor-*γ* coactivator-1*α* (PGC1A) stimulates the expression of genes regulating mitochondrial energy turnover (Mootha *et al*, 2003). We therefore investigated whether PGC1A could be a primary regulator of metabolic pathways that were upregulated in cachectic patients. We chose to focus our analysis on the cluster of 34 PGC1A-responsive OXPHOS genes (OXPHOS-CR subset) identified by Mootha *et al* (2003). Sixteen (64%) of the 25 PGC1A-responsive genes that were included in our GSEA analysis were enriched in cachectic patients. As a comparison, of all OXPHOS genes in the MOOTHA_OXPHOS gene set 62% were enriched in cachectic patients. On our microarrays, mean PGC1A levels in cachectic patients were 171 AU and in controls it was 150 AU. This difference is non-significant with *t*-test. Thus, there is no evidence that cachexia-regulated OXPHOS genes are preferentially regulated by PGC1A, but a role for this co-regulator in controlling fat loss in cancer cachexia cannot be excluded as PGC1A is a common regulator of OXPHOS genes.

The observed upregulation of genes related to electron transport and mitochondria, together with previous reports that FA oxidation is elevated in cachexia, motivated us to analyse adipose tissue mitochondria mass (Agustsson *et al*, 2007). mtDNA copy number per cell did not differ between cachectic *vs* weight-stable patients (values not shown). The levels of the mitochondrial protein ANT was elevated in cachectic (2.0±1.7 AU) *vs* weight-stable patients (0.8±0.8 AU) (*P*=0.03). By contrast, there was no difference in the levels of COX4 protein between cachectic (1.1±0.7 AU) and weight-stable patients (1.3±0.4 AU). Taken together, there was not a consistent increase in mitochondrial OXPHOS proteins; however, an increase in proportion to the general increase in the mRNA levels of mitochondrial/electron transport genes (about 30% Supplementary Table 3) may not easily be detected by immunoblots given the high inter-individual variation.

Systemic inflammation is implicated in human cachexia (Ryden *et al*, 2008); however, there was no evidence for enrichment of inflammatory gene expression among cachectic patients according to the applied microarray analyses.

### Reciprocal regulation of gene expression between cachexia and obesity

We compared the cachexia results with our own previous gene expression profiles from the abdominal subcutaneous adipose tissue of obese and non-obese Swedish women (Dahlman *et al*, 2005). Of the 425 genes that were regulated by cachexia (when using an FDR of 5%), 262 were detected in the analysis of obese *vs* lean individuals. Expression of 83 of the 262 genes was regulated by obesity. More importantly, 82 of the 83 genes were reciprocally regulated by cachexia and obesity and one gene was coherently regulated by cachexia and obesity (a subset of these genes is shown in Table 2). We also compared our results of the cachexia study with those of a French gene expression profiling study of abdominal subcutaneous adipose tissue from 9 obese and 9 lean subjects (Mutch *et al*, 2009). Four hundred and nine of the 425 cachexia-regulated genes were present on the microarray platform used to study obesity in the French cohort; however, only 130 genes were significantly regulated by obesity (FDR=0.5%) in the French cohort. Nevertheless, 105 of these 130 genes were reciprocally regulated by cachexia and obesity. Notably, among the reciprocally regulated genes is the low-density lipoprotein receptor gene, which has been shown to regulate adiposity in experimental models (Goudriaan *et al*, 2001; Yagyu *et al*, 2002).

## Discussion

In this study we have, for the first time, used global gene expression profiling as a tool to unravel regulatory pathways that associate with the wasting of tissues in cancer cachexia. Our cachexia patients had unambiguous signs of cachexia, including self-reported unintentional weight loss, low serum albumin and impaired nutritional status (SGA). Furthermore, the cachectic and control patients had similar lean body mass, which suggests that differences adipose tissue gene expression are not secondary to differences in lean body mass.

Two major pathways associated with cachexia were detected in adipose tissue. First, genes in pathways regulating energy turnover where upregulated, that is, electron transport, FA degradation, oxidative phosphorylation and the Krebs TCA cycle. Second, and more surprising, genes in a number of pathways related to cellular adhesion, extracellular matrix and actin cytoskeleton were downregulated. The changes in the above mentioned pathways are reciprocal to those previously associated with obesity (Dahlman *et al*, 2005; Mutch *et al*, 2009) and similar to the changes observed when obese subjects experienced weight reduction (Henegar *et al*, 2008; Kolehmainen *et al*, 2008). Among all cachexia-regulated genes measured in our own previous obesity studies, the vast majority was reciprocally regulated by obesity and cachexia.

Our findings suggest that regulation of energy turnover, cytoskeleton and extracellular matrix may be important for loss of adipose tissue in cancer patients. The findings for energy turnover are consistent with observations at the whole-body level. Cachexia patients had increased lipid oxidation and enhanced lipid mobilisation from adipose tissue. Fat oxidation in the fat cells could be one of several ways to rid the body of the excess FAs generated by lipolysis. Under normal circumstances the rate of FA oxidation is low in fat cells, but can increase under catabolic conditions (Wang *et al*, 2003). The findings are opposite to those observed in the adipose tissue of obese and type-2 diabetic subjects (Dahlman *et al*, 2006; Henegar *et al*, 2008).

A major function of the extracellular matrix is to provide mechanical support for the cells and all cells need a functional cytoskeleton; however, the matrix components may also participate in a variety of signalling events. Alterations in the extracellular matrix lead to marked metabolic dysregulation and failure to expand adipose tissue during excess caloric intake in rodents (Khan *et al*, 2009). The expression of extracellular matrix genes in human adipose tissue is markedly modified after weight reduction (Kolehmainen *et al*, 2008). In this study we found that cachexia was associated with decreasing fat cell volume, but no change in fat cell number, which is in line with previous results (Agustsson *et al*, 2007). Thus, one possibility is that adipose tissue adjusts its extracellular environment to adapt to the shrinking volume of the fat cells. Such changes in fat cell volume may alter the demand of nutrient and oxygen support by the microvasculature, thereby necessitating an adaptation of the structure of the extracellular matrix. In rodent models of cachexia, WAT is characterised by shrunken adipocytes with dramatically reduced cell size and dilated interstitial space (Bing *et al*, 2006). Furthermore, the shrinking fat cell may have to adjust its cytoskeleton to maintain its intracellular volume to account for the remaining lipid droplet. TREs for HNF4 were overrepresented in the promoter regions of the extracellular matrix and adhesion molecule genes. HNF4 is an important regulator of adhesion proteins (Battle *et al*, 2006). We observed moderately higher mRNA levels of HNF4A in the adipose tissue of weight-stable as compared with that in cachectic patients, which suggests that HNF4 could be a regulator of adhesion molecule gene expression in cachexia.

The observation that cancer cachexia-associated changes in several gene pathways are reciprocal to obesity but similar to those in weight reduction has several implications. First, they may relate to regulation of fat mass, which is not specific to cachexia *per se*. Second, it seems unlikely that tumour-derived factors are responsible for the changes in the above mentioned gene pathways.

Systemic inflammation is believed to be important in cancer cachexia (Deans and Wigmore, 2005; Durham *et al*, 2009). There was no change in the expression of inflammatory genes, suggesting that subcutaneous adipose tissue is not the source for the increased systemic inflammatory activity observed in these cachexia patients (Ryden *et al*, 2008).

In summary, loss of adipose tissue in cancer cachexia is accompanied by marked changes in global gene expression. Most prominent are changes in the pathways regulating energy turnover, extracellular matrix and cytoskeleton, which could have a role in adipose tissue wasting. The gene pathways altered in adipose tissue are in many ways a mirror image to those observed with obesity, but similar to those seen with intentional weight loss, suggesting that regulation of fat mass in cachexia and obesity could, at least in part, be two sides of the same coin.

## Figures and Tables

**Table 1:** Characteristics of study groups

**Measure**	**Cancer – cachexia**	**Cancer – weight-stable**	***P*-value**
Gender, M/F	10/3	9/5	0.52
Age, years	65±6	65±8	0.75
Body mass index (BMI), kg m^−2^	22.4±2.9	25.5±3.6	0.019
Body fat, %	18.5±5.8	27.4±6.6	0.001
Body fat, kg	12.7±4.7	21.0±5.0	<0.001
Lean body mass, kg	57.1±13.5	57.1±14.1	0.99
Weight loss, % of habitual weight	9.7±4.0	0.6±3.1	<0.001
P-glucose, mmol l^−1^	6.6±1.6	6.4±1.7	0.71
P-triglycerides, mmol l^−1^	1.1±0.4	1.4±0.5	0.059
P-cholesterol, mmol l^−1^	4.6±1.3	5.0±1.2	0.41
S-albumin, g l^−1^	34.6±3.9	38.2±2.7	0.012
S-CRP, mg l^−1^	23.5±36.1	2.2±1.8	0.002
S-interleukin-6, *μ*g l^−1^	9.8±8.5	3.5±1.4	0.014
P-glycerol, *μ*mol/l/kg body fat	7.0±4.3	3.4±1.6	0.01
P-fatty acids, mmol/l/kg fat	0.08±0.04	0.04±0.02	0.004
SGA score, points	8 (3–17)	2 (1–3)	<0.001
Tumour stage, points	3 (1–4)	4 (0–4)	0.04
Respiratory quotient, VO_2_/VCO_2_	0.805±0.028	0.849±0.065	0.036
Resting energy expenditure, kcal day^−1^	1639±304	1654±293	0.89
Fat cell volume, pico litres	331±137	504±146	0.006
Fat cell number × 10^10^	4.8±1.7	4.6±0.9	0.78

Abbreviations: P=fasting plasma; S=fasting serum; SGA=Subjective Global Assessment.

Values are mean±s.d. or mean (range). CRP was log_10_ transformed before comparison. Groups were compared by unpaired *t*-test, *χ*^2^-test (for gender) or Mann–Whitney (for SGA and tumour stage).

**Table 2:** Comparison of WAT genes regulated by cachexia with those regulated by obesity^a^

				**Sweden**	**France**	
**Gene name**	**Gene ID**	**Probe set**	**Cachexia/control ratio**	**Lean/obese ratio**		**Adipocyte^b^**
Secreted frizzled-related protein-4	SFRP4	8 139 087	0.32	0.52		0
Sperm-associated antigen-17	SPAG17	7 918 973	0.35			1
RAN-binding protein-3-like	RANBP3 L	8 111 569	0.38			
Atrionatriuretic peptide receptor-C	NPR3	8 104 746	0.40		0.77	1
Secreted frizzled-related protein-2	SFRP2	8 103 254	0.49		0.29	0
Very-low-density lipoprotein receptor	VLDLR	8 154 100	0.52	0.72		1
NAD(P)H dehydrogenase, quinine-1	NQO1	8 002 303	0.53	0.54		1
Vestigial-like-3	VGLL3	8 088 979	0.54			1
S100 calcium-binding protein-A4	S100A4	7 920 271	0.55		0.42	0
Semaphorin-3C	SEMA3C	8 140 534	0.55	0.84	0.43	1
Hypothetical LOC401491	FLJ35024	8 159 850	0.56			1
Semaphorin-3B	SEMA3B	8 079 966	0.56			
Growth factor receptor-bound protein-14	GRB14	8 056 327	0.57			1
Adenylate cyclase-associated protein-2	CAP2	8 117 054	0.58		0.65	1
Follistatin-like-3	FSTL3	8 023 995	0.58	0.73		
Placenta-specific-9	PLAC9	7 928 679	0.58			
Cyclin-D2	CCND2	7 953 200	0.59	0.71	0.73	0
ADAM metallopeptidase domain-22	ADAM22	8 133 983	0.60			0
Leptin (obesity homologue, mouse)	LEP	8 135 909	0.61		0.33	
Peptidylglycine *α*-amidating monooxygenase COOH-terminal interactor	PAMCI	7 965 226	0.61			1
Microtubule-associated protein-1B	MAP1B	8 106 098	0.62			
Solute carrier family-24	SLC24A3	8 061 227	0.62		0.55	
Tripartite motif-containing-16-like	TRIM16 L	8 005 475	0.63			0
Tubulin-*β*-2A	TUBB2A	8 123 644	0.63	0.72	0.37	
Crystallin-*α*B	CRYAB	7 951 662	0.63		0.43	1
Hexamethylene bis-acetamide inducible-1	HEXIM1	8 007 745	0.63	0.73		0
Nexilin (F-actin-binding protein)	NEXN	7 902 495	0.64			1
FRAS1-related extracellular matrix-1	FREM1	8 160 168	0.64		0.63	1
RAB30, member RAS oncogene family	RAB30	7 950 743	0.64			1
Slit homologue-2	SLIT2	8 094 301	0.64	0.70		0
Angiopoietin-1	ANGPT1	8 152 297	0.65	0.77		
Fibroblast growth factor-1	FGF1	8 114 805	0.66	0.66	0.84	1
Ankyrin repeat and death domain-containing-1A	ANKDD1A	7 984 227	0.66			
Family with sequence similarity, 26, member-E	FAM26E	8 121 601	0.67			
Sulphotransferase family, cytosolic, 1A, member-2	SULT1A2	8 000 582	0.67	0.61	0.57	
Growth differentiation factor-10	GDF10	7 933 372	0.67	0.62	1.35	0
WNT1-inducible signalling pathway protein-2	WISP2	8 062 864	0.67	0.63	0.62	0
Hypothetical protein FLJ38359	FLJ38359	8 054 517	0.67			
Tripartite motif-containing-16	TRIM16	8 012 953	0.67		0.37	
Pleckstrin homology domain containing, family Q m.-1	PLEKHQ1	7 984 217	0.68		0.52	0
Midline-1 (Opitz/BBB syndrome)	MID1	8 171 297	0.68	0.75		1
Latent transforming growth factor-*β*-binding protein-2	LTBP2	7 980 152	0.69	0.54	1.21	0
Chromosome-1 open reading frame 198	C1orf198	7 924 996	0.69		0.60	1
Tuftelin-1	TUFT1	7 905 428	0.71			0
Spectrin, *α*, non-erythrocytic-1	SPTAN1	8 158 317	0.71	0.85		0
DIX domain containing-1	DIXDC1	7 943 803	0.71			1
Phosphodiesterase-4D-interacting protein	PDE4DIP	7 919 168	0.71			
Aquaporin-3 (Gill blood group)	AQP3	8 160 670	0.71			
Synaptopodin	SYNPO	8 109 305	0.73		0.58	1
EH domain-binding protein-1	EHBP1	8 042 223	0.73		0.83	
HtrA serine peptidase-1	HTRA1	7 931 097	0.74	0.76	0.37	
Tumour necrosis factor receptor superfamily, m.-25	TNFRSF25	7 912 040	0.75		0.48	
SH3 and PX domain containing-3	SH3PX3	7 985 016	0.76			1
GRB2-related adaptor protein-like	LOC400581	8 005 549	0.76			
Plexin-A1	PLXNA1	8 082 314	0.77			0
Thrombospondin, type-I, domain containing-1	THSD1	7 971 813	0.77			
Actinin, *α*-1	ACTN1	7 979 824	0.77	0.77		1
Sine oculis-binding protein homologue	SOBP	8 121 319	0.77			
ST6 (*α*-*N*-acetyl-neuraminyl-2,3-*β*-galactosyl-1,3)- *N*-acetylgalactosaminide *α*-2,6-sialyltransferase-1	ST6GALNAC1	8 018 774	0.77			
Chromosome-14 open reading frame 4	C14orf4	7 980 338	0.78			0
Plasticity related gene-1	LPPR4	7 903 214	0.78		1.33	0
Notch homolog-3	NOTCH3	8 034 940	0.79	0.70		0
Opioid growth factor receptor-like-1	OGFRL1	8 120 602	0.79		0.62	0
Protein tyrosine phosphatase, receptor type, U	PTPRU	7 899 562	0.79			1
Fatty acid desaturase-3	FADS3	7 948 630	0.79		0.52	0
Rho guanine nucleotide-exchange factor-10-like	ARHGEF10 L	7 898 483	0.82			0
Transient receptor potential cation channel	TRPM4	8 030 251	0.83			1
Zinc finger and BTB domain containing-47	ZBTB47	8 079 099	0.84			
Armadillo repeat containing-7	ARMC7	8 009 755	0.84		1.51	0
Squalene epoxidase	SQLE	8 148 280	0.85			1
Potassium channel tetramerisation domain containing-11	KCTD11	8 004 360	0.85			
Cytochrome-*c* oxidase subunit-8A	COX8A	7 940 835	1.22	1.22		1
Branched chain aminotransferase-2	BCAT2	8 038 202	1.32			
Alcohol dehydrogenase-1C	ADH1C	8 101 893	1.33	1.95	2.20	1
Phosphomannomutase-1	PMM1	8 076 355	1.64	1.53		1
Chromosome-7 open reading frame-24	C7orf24	8 138 857	1.94			1

Abbreviation: WAT=white adipose tissue.

a Results for all cohorts are based on SAM with an FDR of 1% (cachexia and Swedish obesity cohort) or 0.5% (French obesity cohort).

b Genes enriched in fat cells (1) or the stroma vascular fraction (SVF) (0) of white adipose tissue.

**Table 3:** Confirmation of microarray results with RT-qPCR

	**Array cachexia/control^a^**	**RT-qPCR cachexia/control^a^**	***P*-value^b^**	**Array lean/obese^c^**	**RT-qPCR lean/obese^d^**	***P*-value^b^**	**Cell(1)/SVF(0)^e^**
*Cell adhesion*							
ITGB5	0.76	0.70	0.00021	0.62	0.65	0.0001	0
LOXL2	0.69	0.58	0.031	0.79	0.69	0.068	1
MFAP4	0.68	0.63	0.0089	0.57	0.65	0.01	0
ACTN1	0.77	0.55	0.0014	0.77	0.81	0.02	1
ECM2	0.78	0.64	0.0031		1.04	0.37	1
DPT	0.64	0.46	0.0013		1.04	0.42	1
							
*Extracellular matrix*							
LTBP2^f^	0.69	0.54	0.000044	0.54	0.36	0.0003	0
FBLN1	0.69	0.60	0.0042		0.75	0.085	0
CILP	0.63	0.66	0.028		0.33	0.0031	0
							
*Actin cytoskeleton*							
SPTAN1	0.71	0.64	0.0018	0.85	0.83	0.12	0
							
*Mitochondrion*							
COX8a^f^	1.22	1.50	0.0079	1.22	1.00	0.49	1
CYC1	1.24	1.40	0.0076	1.21	1.06	0.30	1
PC	1.45	1.90	0.00027	1.38	1.42	0.0017	1

Abbreviation: RT-qPCR=real-time quantitative PCR.

a Cachexia (*n*=13), control (*n*=14).

b *t*-test, one-sided.

c Non-obese (*n*=17) and 20 obese (*n*=20).

d Non-obese (*n*=13) and 20 obese (*n*=19).

e Genes enriched in fat cells (1) or the stroma vascular fraction (SVF) (0) of white adipose tissue.

f Significant difference on microarrays with FDR 1% between cachectic and weight-stable patients. Remaining genes are significant with an FDR of 5%.

**Table 4:** Pathways regulated by cachexia in adipose tissue according to GSEA

**Pathways**	**No. of genes**	**Nominal P**	**FDR *q*-value**
*Enriched in cachexia*			
ELECTRON_TRANSPORT_CHAIN	98	0.008	0.19
STATIN_PATHWAY_PHARMGKB	18	0.002	0.15
FATTY_ACID_DEGRADATION	23	0.004	0.17
MOOTHA_VOXPHOS	77	0.025	0.14
OXIDATIVE_PHOSPHORYLATION	59	0.041	0.17
FATTY_ACID_SYNTHESIS	16	0.018	0.16
KREBS_TCA_CYCLE	31	0.012	0.16
ADIPOCYTE_PPARG_UP	16	0.021	0.14
UBIQUINONE_BIOSYNTHESIS	15	0.002	0.14
HUMAN_MITODB_6_2002	379	0.029	0.13
NADLER_OBESITY_DN	36	0.028	0.12
ALANINE_AND_ASPARTATE_ METABOLISM	21	0.022	0.12
HYPOPHYSECTOMY_RAT_UP	34	0.03	0.13
TNFALPHA_TGZ_ADIP_DN	28	0.046	0.15
CITRATE_CYCLE_TCA_CYCLE	19	0.022	0.15
TCA	15	0.004	0.14
MITOCHONDRIA	387	0.051	0.14
FATTY_ACID_METABOLISM	79	0.05	0.17
			
*Enriched in controls*			
PASSERINI_EM	34	0	0.19
SCHRAETS_MLL_UP	33	0	0.17

Abbreviations: FDR=false discovery rate; GSEA=Gene Set Enrichment Analysis.

Shown are pathways with an FDR *q*-value <0.20.
