# Supplementary material for: Adipose tissue pathways involved in weight loss of cancer cachexia
Source: Br J Cancer. 2010 Apr 20;102(10):1541–8. doi: 10.1038/sj.bjc.6605665 (PMC2869165; doi:10.1038/sj.bjc.6605665)
Supplement: Supplementary methods [file 6605665x1.doc]

**Supplementary methods**

**Power calculation**

We used the method by Liu to determine the appropriate sample size for microarray experiments (Liu & Hwang, 2007). Assuming that significant differences in gene expression between the two studied groups were defined with a false discovery rate (FDR) of 5%, the proportion of non-differentially expressed genes between groups was 0.95. Assuming a standard deviation for effect size of 1, a population consisting of two groups composed of 15 subjects each yields a power of approximately 90% to detect differentially expressed genes. This is close to the actual number of 13 cachectic and 14 weight stable patients used in the present analysis.

**Quantification of mtDNA copy number**

Briefly, a 120 nucleotide-long mtDNA fragment within the mitochondrial NADH dehydrogenase subunit 1 (ND1) gene was used for quantification of mtDNA. A 120 bp region of the nuclear gene lipoprotein lipase (LPL) was used to normalize results. Plasmid standard curves of known concentration containing the ND1 and LPL fragment were used to determine ND1 copy numbers per cell of studied samples.

**Protein expression**

Total protein lysates were obtained from approximately 300 mg of white adipose tissue, as previously described (Arner et al, 2008) . One hundred micrograms of total cellular protein was loaded and separated by SDS-PAGE. Gels were transferred to polyvinylidine fluoride membranes (Amersham Pharmacia, Little Chaffore, UK). Blots were blocked in Tris-buffered saline with 0.1% Tween-20 and 5% non-fat dried milk. This was followed by an overnight incubation at 4^o^C in the presence of antibodies directed against Adenine nucleotide translocator (ANT) and Cytochrome C oxidase subunit 4 (COX-4) (both from Santa Cruz, CA) or the control protein β-actin (SIGMA, St Louis, USA). Secondary antibodies were from Sigma. Antigen-antibody complexes were detected by chemiluminescence using a detection kit from Cell Signaling (LumiGlo, Boston, MA) and specific bands were detected and quantified using a Chemidoc XRS system and the Quantity One Software (Bio-Rad), and expressed as a ratio to β-actin in the same sample.

| **Supplementary table 1** |  |  |  |  |  |  |
| --- | --- | --- | --- | --- | --- | --- |

**White adipose tissue expressed genes regulated by cachexia***

| **Gene id** | **Probeset** | **chachexia (N=13)/**  **controls (N=14)†** |  | **Gene id** | **Probeset** | **chachexia (N=13)/**  **controls (N=14)†** |
| --- | --- | --- | --- | --- | --- | --- |
| ABCC1 | 7993478 | 0.79 |  | IGFBP6 | 7955694 | 0.68 |
| ACBD4 | 8007730 | 0.81 |  | IMP3 | 7990540 | 0.89 |
| ACTN1 | 7979824 | 0.77 |  | IMPAD1 | 8150906 | 0.71 |
| ADAM22 | 8133983 | 0.60 |  | INS | 7945688 | 0.72 |
| ADAM28 | 8145293 | 0.77 |  | INTS7 | 7924119 | 0.76 |
| ADAMTS10 | 8033635 | 0.68 |  | IRF2BP2 | 7925161 | 0.84 |
| ADAMTS2 | 8116272 | 0.77 |  | IRX6 | 7995674 | 0.71 |
| ADCK1 | 7976000 | 0.76 |  | ITGA3 | 8008237 | 0.79 |
| ADCY7 | 7995492 | 0.73 |  | ITGA9 | 8078619 | 0.80 |
| AFAP1L2 | 7936439 | 0.78 |  | ITGB5 | 8090162 | 0.76 |
| AFF3 | 8054254 | 0.66 |  | ITIH5 | 7931977 | 0.73 |
| AFMID | 8010248 | 0.84 |  | ITPK1 | 7980970 | 0.80 |
| AGTRL1 | 7948167 | 0.54 |  | KCNB1 | 8066921 | 0.77 |
| AHNAK | 7948667 | 0.84 |  | KCNT2 | 7923043 | 0.69 |
| AKR1C1 | 7925918 | 0.78 |  | KCTD11 | 8004360 | 0.85 |
| AKR1C2 | 7931832 | 0.77 |  | KIAA0284 | 7977299 | 0.81 |
| ALDH1A2 | 7989199 | 0.75 |  | KIAA1026 | 7898084 | 0.80 |
| AMOT | 8174576 | 0.65 |  | KIAA1467 | 7954077 | 0.86 |
| AMOTL2 | 8090852 | 0.72 |  | KIAA1602 | 7963121 | 0.67 |
| ANGPT1 | 8152297 | 0.65 |  | KIAA1706 | 8132305 | 0.73 |
| ANKDD1A | 7984227 | 0.66 |  | KIF1C | 8004057 | 0.79 |
| ANKRD38 | 7916654 | 0.61 |  | KIF5C | 8045637 | 0.79 |
| AOC2 | 8007414 | 0.79 |  | KLF10 | 8152215 | 0.75 |
| AQP3 | 8160670 | 0.71 |  | KLHL30 | 8049610 | 0.65 |
| ARHGAP20 | 7951565 | 0.67 |  | KLHL31 | 8127177 | 0.63 |
| ARHGAP21 | 7932554 | 0.73 |  | LACE1 | 8121349 | 0.78 |
| ARHGAP28 | 8019964 | 0.76 |  | LAMB2 | 8087337 | 0.79 |
| ARHGEF10 | 8144281 | 0.76 |  | LEP | 8135909 | 0.61 |
| ARHGEF10L | 7898483 | 0.82 |  | LFNG | 8131143 | 0.74 |
| ARMC7 | 8009755 | 0.84 |  | LHCGR | 8052058 | 0.54 |
| ASAM | 7952341 | 0.78 |  | LOC162073 | 7993622 | 0.77 |
| ASPH | 8150988 | 0.80 |  | LOC349196 | 8149216 | 0.76 |
| AXIN2 | 8017718 | 0.62 |  | LOC400581 | 8005549 | 0.76 |
| B4GALT2 | 7900931 | 0.87 |  | LOC401233 | 8123637 | 0.70 |
| B4GALT6 | 8022747 | 0.56 |  | LOC541471 | 8054611 | 0.79 |
| BACE2 | 8068671 | 0.82 |  | LOC729278 | 8144492 | 0.77 |
| BAG3 | 7930921 | 0.80 |  | LOX | 8113709 | 0.57 |
| BHMT2 | 8106494 | 0.72 |  | LOXL2 | 8149774 | 0.69 |
| BICD1 | 7954717 | 0.83 |  | LOXL3 | 8053231 | 0.75 |
| BMF | 7987454 | 0.83 |  | LOXL4 | 7935553 | 0.79 |
| BTBD14A | 8165077 | 0.86 |  | LPGAT1 | 7924107 | 0.74 |
| C10orf11 | 7928534 | 0.78 |  | LPPR4 | 7903214 | 0.78 |
| C10orf38 | 7932243 | 0.80 |  | LRRFIP1 | 8049544 | 0.75 |
| C14orf4 | 7980338 | 0.78 |  | LTBP2 | 7980152 | 0.69 |
| C14orf43 | 7980051 | 0.82 |  | LTBP3 | 7949412 | 0.79 |
| C17orf28 | 8018264 | 0.81 |  | MAGED1 | 8167656 | 0.81 |
| C18orf10 | 8022941 | 0.80 |  | MALL | 8054479 | 0.73 |
| C1orf164 | 7900979 | 0.86 |  | MAP1B | 8106098 | 0.62 |
| C1orf198 | 7924996 | 0.69 |  | MASP1 | 8092661 | 0.59 |
| C20orf117 | 8066091 | 0.88 |  | MFAP4 | 8013341 | 0.68 |
| C22orf9 | 8076690 | 0.76 |  | MICAL3 | 8074274 | 0.85 |
| C2orf59 | 8043363 | 0.77 |  | MID1 | 8171297 | 0.68 |
| C4orf31 | 8102587 | 0.68 |  | MLPH | 8049487 | 0.74 |
| C6orf105 | 8123951 | 0.78 |  | MLSTD1 | 7954631 | 0.75 |
| C6orf138 | 8126853 | 0.58 |  | MMP28 | 8014282 | 0.74 |
| C6orf145 | 8123678 | 0.78 |  | MN1 | 8075126 | 0.78 |
| C9orf100 | 8161026 | 0.81 |  | MSC | 8151334 | 0.54 |
| CALU | 8135955 | 0.72 |  | MSTO1 | 7906021 | 0.64 |
| CAMK2G | 7934477 | 0.81 |  | MUC3B | 8135015 | 0.78 |
| CAP2 | 8117054 | 0.58 |  | MYCBP | 7915170 | 0.89 |
| CASP10 | 8047403 | 0.78 |  | NAT11 | 7940824 | 0.80 |
| CAV1 | 8135594 | 0.76 |  | NAV3 | 7957298 | 0.69 |
| CCDC102B | 8021685 | 0.75 |  | NEXN | 7902495 | 0.64 |
| CCDC80 | 8089544 | 0.65 |  | NOTCH3 | 8034940 | 0.79 |
| CCND2 | 7953200 | 0.59 |  | NPHP1 | 8054486 | 0.78 |
| CD109 | 8120719 | 0.76 |  | NPR3 | 8104746 | 0.40 |
| CD1C | 7906348 | 0.62 |  | NPY1R | 8103494 | 0.59 |
| CD200 | 8081657 | 0.77 |  | NQO1 | 8002303 | 0.53 |
| CD248 | 7949588 | 0.65 |  | NRP2 | 8047738 | 0.73 |
| CD28 | 8047677 | 0.62 |  | ODZ4 | 7950701 | 0.71 |
| CD34 | 7923978 | 0.79 |  | OGFRL1 | 8120602 | 0.79 |
| CD3EAP | 8029688 | 0.83 |  | OLFML2B | 7921882 | 0.67 |
| CD9 | 7953291 | 0.74 |  | P2RX7 | 7959251 | 0.75 |
| CDC42EP1 | 8072817 | 0.78 |  | P4HA2 | 8113981 | 0.78 |
| CDK6 | 8140955 | 0.74 |  | PALLD | 8098263 | 0.67 |
| CDKN2B | 8160452 | 0.63 |  | PAMCI | 7965226 | 0.61 |
| CES1 | 8001457 | 0.61 |  | PARD3B | 8047709 | 0.77 |
| CES4 | 7995729 | 0.57 |  | PDE4DIP | 7919168 | 0.71 |
| CFH | 7908459 | 0.61 |  | PDGFA | 8137670 | 0.80 |
| CFHR1 | 7908488 | 0.58 |  | PEA15 | 7906564 | 0.82 |
| CFHR3 | 7908481 | 0.59 |  | PHF19 | 8163807 | 0.87 |
| CHMP7 | 8145259 | 0.81 |  | PLAC9 | 7928679 | 0.58 |
| CHST7 | 8166999 | 0.84 |  | PLCD3 | 8016168 | 0.74 |
| CHSY-2 | 8107850 | 0.77 |  | PLEKHG5 | 7912056 | 0.64 |
| CILP | 7989750 | 0.63 |  | PLEKHQ1 | 7984217 | 0.68 |
| CLEC3B | 8079305 | 0.72 |  | PLK2 | 8112202 | 0.59 |
| CLIP3 | 8036252 | 0.86 |  | PLXNA1 | 8082314 | 0.77 |
| CLN8 | 8144267 | 0.83 |  | PPP1R9B | 8016628 | 0.82 |
| CLPTM1L | 8110803 | 0.83 |  | PTPRU | 7899562 | 0.79 |
| CNN3 | 7917885 | 0.82 |  | RAB11FIP3 | 7991860 | 0.87 |
| COBLL1 | 8056343 | 0.73 |  | RAB30 | 7950743 | 0.64 |
| COL11A1 | 7918064 | 0.81 |  | RAMP3 | 8132660 | 0.74 |
| COL12A1 | 8127563 | 0.71 |  | RANBP3L | 8111569 | 0.38 |
| COL15A1 | 8156783 | 0.76 |  | RASA3 | 7972946 | 0.70 |
| COL16A1 | 7914361 | 0.82 |  | S100A4 | 7920271 | 0.55 |
| COL5A2 | 8057620 | 0.76 |  | S100A6 | 7920258 | 0.72 |
| COL6A1 | 8069269 | 0.81 |  | SAMD4A | 7974425 | 0.67 |
| COL9A2 | 7915297 | 0.83 |  | SCIN | 8131550 | 0.44 |
| CORO1C | 7966135 | 0.76 |  | SEMA3B | 8079966 | 0.56 |
| CPNE2 | 7995976 | 0.83 |  | SEMA3C | 8140534 | 0.55 |
| CREB3 | 8155096 | 0.85 |  | septin5 | 8071268 | 0.86 |
| CRY1 | 7966052 | 0.78 |  | septin8 | 8114050 | 0.82 |
| CRYAB | 7951662 | 0.63 |  | SFRP2 | 8103254 | 0.49 |
| CSF1 | 7903786 | 0.79 |  | SFRP4 | 8139087 | 0.32 |
| CTGF | 8129562 | 0.68 |  | SH2D4A | 8144880 | 0.71 |
| CUTC | 7929768 | 0.79 |  | SH3BP4 | 8049435 | 0.86 |
| CXCL2 | 8100994 | 0.69 |  | SH3PX3 | 7985016 | 0.76 |
| CXXC5 | 8108447 | 0.76 |  | SIPA1L1 | 7975459 | 0.72 |
| CYB561 | 8017378 | 0.82 |  | SLC24A3 | 8061227 | 0.62 |
| CYBASC3 | 7948565 | 0.77 |  | SLC39A4 | 8153762 | 0.84 |
| CYSLTR2 | 7969050 | 0.69 |  | SLIT2 | 8094301 | 0.64 |
| DAAM1 | 7974697 | 0.78 |  | SNCG | 7928872 | 0.67 |
| DAB2IP | 8157610 | 0.73 |  | SOBP | 8121319 | 0.77 |
| DAG1 | 8079753 | 0.84 |  | SORBS3 | 8145151 | 0.85 |
| DAGLA | 7940508 | 0.84 |  | SPAG17 | 7918973 | 0.35 |
| DBN1 | 8116051 | 0.78 |  | SPECC1 | 8005661 | 0.80 |
| DCHS1 | 7946245 | 0.86 |  | SPTAN1 | 8158317 | 0.71 |
| DCUN1D3 | 8000028 | 0.80 |  | SPTBN1 | 8041995 | 0.81 |
| DDAH2 | 8178590 | 0.83 |  | SQLE | 8148280 | 0.85 |
| DDEF2 | 8040113 | 0.80 |  | ST6GALNAC1 | 8018774 | 0.77 |
| DDR2 | 7906878 | 0.80 |  | STK40 | 7914904 | 0.80 |
| DGKI | 8143154 | 0.68 |  | SULT1A2 | 8000582 | 0.67 |
| DIXDC1 | 7943803 | 0.71 |  | SYDE1 | 8026407 | 0.83 |
| DLGAP4 | 8062293 | 0.86 |  | SYNPO | 8109305 | 0.73 |
| DMN | 7986385 | 0.76 |  | THSD1 | 7971813 | 0.77 |
| DMPK | 8037657 | 0.79 |  | TMCO3 | 7970301 | 0.81 |
| DNAJB5 | 8154962 | 0.84 |  | TMEM30B | 7979524 | 0.74 |
| DNMT1 | 8033912 | 0.85 |  | TMEM64 | 8151747 | 0.71 |
| DOK1 | 8042917 | 0.79 |  | TMEPAI | 8067233 | 0.58 |
| DPT | 7922130 | 0.64 |  | TNFRSF25 | 7912040 | 0.75 |
| DPYSL3 | 8114920 | 0.78 |  | TNMD | 8168737 | 0.48 |
| DTX2 | 8133736 | 0.78 |  | TNXB | 8125234 | 0.73 |
| DUSP10 | 7924450 | 0.66 |  | TPM2 | 8161044 | 0.79 |
| DUSP14 | 8006736 | 0.74 |  | TRIM16 | 8012953 | 0.67 |
| DYNC1I1 | 8134384 | 0.87 |  | TRIM16L | 8005475 | 0.63 |
| DZIP1L | 8090938 | 0.72 |  | TRPM4 | 8030251 | 0.83 |
| ECM2 | 8162404 | 0.78 |  | TTC7A | 8041826 | 0.80 |
| ECOP | 7904969 | 0.79 |  | TTLL1 | 8076547 | 0.87 |
| EEA1 | 7965436 | 0.76 |  | TUBB2A | 8123644 | 0.63 |
| EFEMP1 | 8052355 | 0.78 |  | TUFT1 | 7905428 | 0.71 |
| EHBP1 | 8042223 | 0.73 |  | UCHL1 | 8094778 | 0.43 |
| ELA3B | 7898713 | 0.88 |  | VASH1 | 7975889 | 0.74 |
| ELN | 8133372 | 0.76 |  | VASN | 7992967 | 0.79 |
| ENC1 | 8112615 | 0.72 |  | VGLL3 | 8088979 | 0.54 |
| EPDR1 | 8132369 | 0.69 |  | VLDLR | 8154100 | 0.52 |
| ERBB2 | 8006906 | 0.80 |  | WDR54 | 8042843 | 0.83 |
| ETV3 | 7921228 | 0.79 |  | WISP2 | 8062864 | 0.67 |
| F2R | 8106393 | 0.65 |  | YAP1 | 7943398 | 0.81 |
| FADS3 | 7948630 | 0.79 |  | ZBTB47 | 8079099 | 0.84 |
| FAM102B | 7903507 | 0.73 |  | ZFAT1 | 8153043 | 0.78 |
| FAM129B | 8164217 | 0.80 |  | ZNF219 | 7977646 | 0.68 |
| FAM26E | 8121601 | 0.67 |  | AASS | 8142554 | 1.48 |
| FAM69B | 8159373 | 0.84 |  | ABCA1 | 8162940 | 1.43 |
| FAM84B | 8152812 | 0.79 |  | ABHD5 | 8079153 | 1.68 |
| FAT | 8104079 | 0.71 |  | ADH1A | 8101874 | 1.39 |
| FAT2 | 8115302 | 0.66 |  | ADH1B | 8101881 | 1.37 |
| FAT4 | 8097288 | 0.74 |  | ADH1C | 8101893 | 1.33 |
| FBLN1 | 8073775 | 0.69 |  | AGT | 7924987 | 1.57 |
| FCER1A | 7906443 | 0.55 |  | AK3L1 | 7902038 | 1.40 |
| FCGBP | 8036787 | 0.51 |  | APOE | 8029530 | 1.77 |
| FGD5 | 8078066 | 0.78 |  | BCAT2 | 8038202 | 1.32 |
| FGF1 | 8114805 | 0.66 |  | C20orf3 | 8065433 | 1.37 |
| FGF11 | 8004408 | 0.84 |  | C21orf15 | 8069505 | 1.94 |
| FIBIN | 7939052 | 0.71 |  | C7orf24 | 8138857 | 1.94 |
| FKBP14 | 8138834 | 0.72 |  | CABC1 | 7910164 | 1.54 |
| FKBP9 | 8132214 | 0.85 |  | CALCRL | 8057578 | 1.57 |
| FLJ35024 | 8159850 | 0.56 |  | CDC34 | 8023968 | 1.22 |
| FLJ36031 | 8142136 | 0.86 |  | CKB | 7981427 | 1.60 |
| FLJ37543 | 8105517 | 0.79 |  | CNTFR | 8160823 | 1.29 |
| FLJ38359 | 8054517 | 0.67 |  | COX8A | 7940835 | 1.22 |
| FLJ40869 | 8040440 | 0.79 |  | CRLS1 | 8060839 | 1.41 |
| FLJ41603 | 8109161 | 0.78 |  | CYB5A | 8023855 | 1.33 |
| FLJ44796 | 8114006 | 0.72 |  | CYC1 | 8148728 | 1.24 |
| FLJ45983 | 7932014 | 0.80 |  | DDR1 | 8179184 | 1.39 |
| FMNL2 | 8045736 | 0.75 |  | ECHDC3 | 7926152 | 1.71 |
| FN1 | 8058765 | 0.73 |  | EIF4EBP1 | 8145889 | 1.47 |
| FREM1 | 8160168 | 0.64 |  | EPM2AIP1 | 8086148 | 1.20 |
| FRMD4B | 8088745 | 0.69 |  | GCDH | 8026090 | 1.27 |
| FSCN1 | 8131339 | 0.80 |  | GLUL | 7922689 | 1.51 |
| FSTL3 | 8023995 | 0.58 |  | GPD1L | 8078386 | 1.67 |
| GAS1 | 8162179 | 0.82 |  | GPHN | 7975167 | 1.34 |
| GAS2L3 | 7957850 | 0.68 |  | GPT | 8148902 | 1.40 |
| GATA2 | 8090469 | 0.89 |  | GSDML | 8014903 | 1.99 |
| GCNT2 | 8116835 | 0.75 |  | IGF1 | 7965873 | 1.52 |
| GDA | 8155802 | 0.66 |  | KIAA1772 | 8020384 | 1.64 |
| GDF10 | 7933372 | 0.67 |  | KLF15 | 8090343 | 1.46 |
| GFRA2 | 8149629 | 0.69 |  | LDHD | 8002830 | 1.62 |
| GGH | 8151032 | 0.68 |  | LGALS12 | 7940762 | 1.42 |
| GIMAP7 | 8137240 | 0.79 |  | LOC646119 | 7930919 | 1.28 |
| GNA12 | 8137865 | 0.79 |  | LOC728131 | 8101357 | 1.33 |
| GNG2 | 7974341 | 0.65 |  | LPIN1 | 8040340 | 1.87 |
| GOLGA3 | 7967810 | 0.80 |  | MAG1 | 8096116 | 3.57 |
| GPC1 | 8049670 | 0.75 |  | MAP1LC3C | 7925504 | 1.85 |
| GPER | 8131069 | 0.79 |  | MCCC2 | 8177601 | 1.28 |
| GPLD1 | 8124211 | 0.65 |  | MOCS1 | 8126214 | 1.63 |
| GRB14 | 8056327 | 0.57 |  | MPST | 8072777 | 1.20 |
| GRIN2B | 7961422 | 0.64 |  | MST150 | 8109326 | 1.21 |
| GRINL1A | 7983890 | 0.70 |  | NR1H3 | 7939751 | 1.29 |
| HDAC7A | 7962659 | 0.85 |  | ORMDL3 | 8014916 | 1.64 |
| HDGF | 7921133 | 0.85 |  | PC | 7949719 | 1.45 |
| HEXIM1 | 8007745 | 0.63 |  | PCYT2 | 8019280 | 1.39 |
| HIST1H1A | 8124380 | 0.74 |  | PDE1B | 7955943 | 1.38 |
| HIVEP3 | 7915392 | 0.81 |  | PKP2 | 7962212 | 2.13 |
| HNT | 7945245 | 0.67 |  | PMM1 | 8076355 | 1.64 |
| HOMER3 | 8035566 | 0.86 |  | PTEN | 7928959 | 1.35 |
| HOXC8 | 7955869 | 0.80 |  | PYGM | 7949124 | 1.54 |
| HSD11B1 | 7909446 | 0.49 |  | RGS3 | 8157324 | 1.65 |
| HSPA12A | 7936516 | 0.77 |  | SLC2A1 | 7915472 | 1.39 |
| HSPB2 | 7943787 | 0.85 |  | TARP | 8139100 | 1.79 |
| HSPB8 | 7959102 | 0.66 |  | THYN1 | 7952869 | 1.39 |
| HTRA1 | 7931097 | 0.74 |  | VEGFA | 8119898 | 2.11 |
| HYPE | 7958410 | 0.86 |  | ZFAND5 | 8161747 | 1.55 |
| IGF2 | 7937772 | 0.65 |  |  |  |  |
| *Based on SAM with 5% FDR. †Mean fold change. | | | | |  |  |

| **Supplementary table 2**  **Gene ontologies regulated by cachexia in white adipose tissue^*^** | | | |
| --- | --- | --- | --- |
|  | **No. of regulated genes** | |  |
| **Category** | **Observed** | **Expected** | **P-value** |
| **Down in cachexia** |  |  |  |
| **Biological Process** |  |  |  |
| **cell adhesion** | 30 | 11.7 | 0.0000019 |
| signal transduction | 78 | 60.0 | 0.0048 |
| enzyme linked receptor protein signaling pathway | 14 | 3.7 | 0.000018 |
| transmembrane receptor protein tyrosine kinase signaling | 10 | 2.6 | 0.00026 |
| regulation of small GTPase mediated signal transduction | 8 | 2.3 | 0.0019 |
| regulation of Rho protein signal transduction | 5 | 1.3 | 0.0081 |
| neurite development | 5 | 1.2 | 0.0076 |
| regulation of cell cycle | 18 | 9.1 | 0.0044 |
| **actin cytoskeleton organization and biogenesis** | 14 | 3.0 | 0.0000019 |
| regulation of cell organization and biogenesis | 6 | 1.2 | 0.0011 |
| phosphate transport | 7 | 1.6 | 0.0010 |
| Morphogenesis | 28 | 12.1 | 0.000030 |
| Angiogenesis | 6 | 1.4 | 0.0031 |
| organ development | 22 | 11.3 | 0.0021 |
| skeletal development | 9 | 2.7 | 0.0017 |
| acute inflammatory response | 5 | 1.3 | 0.0099 |
|  |  |  |  |
| **Molecular function** |  |  |  |
| calcium ion binding | 32 | 15.8 | 0.00011 |
| copper ion binding | 6 | 1.2 | 0.0011 |
| growth factor binding | 8 | 1.2 | 0.000028 |
| insulin-like growth factor binding | 6 | 0.5 | 0.0000043 |
| protein complex binding | 6 | 1.3 | 0.0021 |
| electron carrier activity | 9 | 3.3 | 0.0060 |
| oxidoreductase activity acting on CH-NH2 group | 5 | 0.3 | 0.000014 |
|  |  |  |  |
| **Cellular component** |  |  |  |
| Golgi apparatus | 12 | 5.0 | 0.0045 |
| Cytoskeleton | 30 | 16.2 | 0.00075 |
| **extracellular matrix** | 27 | 5.5 | 6E-12 |
|  |  |  |  |
| **Up in cachexia** |  |  |  |
| **Biologicial process** |  |  |  |
| alcohol metabolism | 8 | 1.0 | 0.0000056 |
| monosaccharide metabolism | 4 | 0.5 | 0.0018 |
| hexose metabolism | 4 | 0.5 | 0.0016 |
| amino acid metabolism | 4 | 0.8 | 0.0094 |
| generation of precursor metabolites and energy | 8 | 2.0 | 0.00080 |
| **electron transport** | 5 | 1.2 | 0.0069 |
| organic acid metabolism | 8 | 1.6 | 0.00018 |
| carboxylic acid metabolism | 7 | 1.6 | 0.0010 |
| Metabolism | 35 | 26.2 | 0.0048 |
| cellular carbohydrate metabolism | 5 | 1.2 | 0.0064 |
| nitrogen compound metabolism | 6 | 1.4 | 0.0021 |
|  |  |  |  |
| **Molecular function** |  |  |  |
| catalytic activity | 30 | 15.7 | 1.102E-05 |
| oxidoreductase activity | 8 | 2.3 | 0.0016 |
|  |  |  |  |
| **Cellular component** |  |  |  |
| intracellular part | 32 | 24.3 | 0.0091 |
| Cytoplasm | 23 | 11.5 | 0.00015 |
| **Mitochondrion** | 12 | 2.3 | 1.473E-06 |
| ***** Results from analysis by GOTM based on genes regulated by SAM with 5% FDR. | | | |
| Shown are GO with >3 observed regulated genes. Excluded are GO with highly overlapping content. | | | |

| **Supplementary table 3**  **Genes in a subset of GO regulated by cachexia** | | |
| --- | --- | --- |
|  |  | **chachexia/** |
| **Cell adhesion and extracellular matrix** | **gene id** | **control** |
| protein tyrosine phosphatase, receptor type, U | PTPRU | 0.79 |
| sorbin and SH3 domain containing 3 | SORBS3 | 0.85 |
| collagen, type VI, alpha 1 | COL6A1 | 0.81 |
| collagen, type XI, alpha 1 | COL11A1 | 0.81 |
| collagen, type XII, alpha 1 | COL12A1 | 0.71 |
| collagen, type XV, alpha 1 | COL15A1 | 0.76 |
| collagen, type XVI, alpha 1 | COL16A1 | 0.82 |
| connective tissue growth factor | CTGF | 0.68 |
| FRAS1 related extracellular matrix 1 | FREM1 | 0.64 |
| Dermatopontin | DPT | 0.64 |
| extracellular matrix protein 2 | ECM2 | 0.78 |
| FAT tumor suppressor homolog 1 | FAT | 0.71 |
| FAT tumor suppressor homolog 2 | FAT2 | 0.66 |
| fibronectin 1 | FN1 | 0.73 |
| integrin, alpha 3 | ITGA3 | 0.79 |
| integrin, alpha 9 | ITGA9 | 0.80 |
| integrin, beta 5 | ITGB5 | 0.76 |
| laminin, beta 2 | LAMB2 | 0.79 |
| lysyl oxidase-like 2 | LOXL2 | 0.69 |
| microfibrillar-associated protein 4 | MFAP4 | 0.68 |
| nephronophthisis 1 | NPHP1 | 0.78 |
| discoidin domain receptor family, member 2 | DDR2 | 0.80 |
| ADAM metallopeptidase domain 22 | ADAM22 | 0.60 |
| tenascin XB | TNXB | 0.73 |
| dachsous 1 (Drosophila) | DCHS1 | 0.86 |
| actinin, alpha 1 | ACTN1 | 0.77 |
| neuropilin 2 | NRP2 | 0.73 |
| WNT1 inducible signaling pathway protein 2 | WISP2 | 0.67 |
| CD9 molecule | CD9 | 0.74 |
| CD34 molecule | CD34 | 0.79 |
| collagen, type V, alpha 2 | COL5A2 | 0.76 |
| dystroglycan 1 | DAG1 | 0.84 |
| elastin | ELN | 0.76 |
| fibulin 1 | FBLN1 | 0.69 |
| EGF-containing fibulin-like ECM 1 | EFEMP1 | 0.78 |
| glypican 1 | GPC1 | 0.75 |
| lysyl oxidase | LOX | 0.57 |
| latent transforming growth factor beta binding protein 2 | LTBP2 | 0.69 |
| microfibrillar-associated protein 4 | MFAP4 | 0.68 |
| CD248 molecule, endosialin | CD248 | 0.65 |
| matrix metallopeptidase 28 | MMP28 | 0.74 |
| ADAM metallopeptidase with thrombospondin type 1 motfif, 10 | ADAMTS10 | 0.68 |
| cartilage intermediate layer protein | CILP | 0.63 |
| ADAM metallopeptidase with thrombospondin type 1 motif, 2 | ADAMTS2 | 0.77 |
|  |  |  |
| **Actin cytoskeleton organization and biogenesis** |  |  |
| sorbin and SH3 domain containing 3 | SORBS3 | 0.85 |
| formin-like 2 | FMNL2 | 0.75 |
| calponin 3, acidic | CNN3 | 0.82 |
| FYVE, RhoGEF and PH domain containing 5 | FGD5 | 0.78 |
| Angiomotin | AMOT | 0.65 |
| drebrin 1 | DBN1 | 0.78 |
| dishevelled associated activator of morphogenesis 1 | DAAM1 | 0.78 |
| nephronophthisis 1 | NPHP1 | 0.78 |
| Rho guanine nucleotide exchange factor 10-like | ARHGEF10L | 0.82 |
| fascin homolog 1, actin-bundling protein | FSCN1 | 0.80 |
| spectrin, alpha, non-erythrocytic 1 | SPTAN1 | 0.71 |
| spectrin, beta, non-erythrocytic 1 | SPTBN1 | 0.81 |
| Scinderin | SCIN | 0.44 |
| phosphodiesterase 4D interacting protein | PDE4DIP | 0.71 |
|  |  |  |
| **Mitochondrion and electron transport genes** |  |  |
| aminoadipate-semialdehyde synthase | AASS | 1.48 |
| cytochrome c oxidase subunit 8A | COX8A | 1.22 |
| cytochrome b5 type A | CYB5A | 1.33 |
| cytochrome c-1 | CYC1 | 1.24 |
| adenylate kinase 3-like 1 | AK3L1 | 1.40 |
| glutaryl-Coenzyme A dehydrogenase | GCDH | 1.27 |
| mercaptopyruvate sulfurtransferase | MPST | 1.20 |
| pyruvate carboxylase | PC | 1.45 |
| cardiolipin synthase 1 | CRLS1 | 1.41 |
| chaperone, ABC1 activity of bc1 complex | CABC1 | 1.54 |
| branched chain aminotransferase 2 | BCAT2 | 1.32 |
| methylcrotonoyl-Coenzyme A carboxylase 2 | MCCC2 | 1.28 |
| Cell adhesion and extracellular matrix genes, as well as mitochondria and electron transport genes are shown together due to overlap between GOs, | | |

Supplementary figure 1


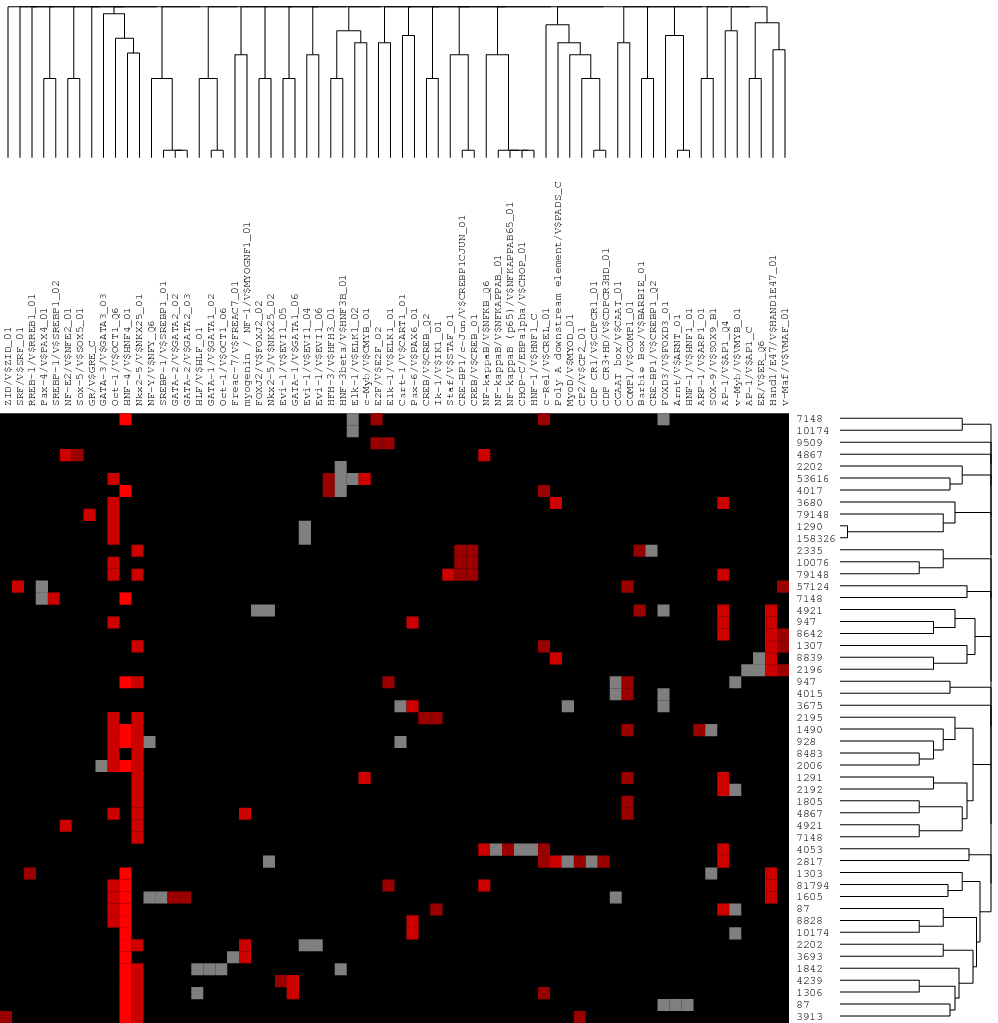


Figures on the Y-axis represent Entrez gene IDs for extracellular matrix and cell adhesion genes.
